# An Exploratory Study to Detect Ménière’s Disease in Conventional MRI Scans Using Radiomics

**DOI:** 10.3389/fneur.2016.00190

**Published:** 2016-11-07

**Authors:** E. L. van den Burg, M. van Hoof, A. A. Postma, A. M. L. Janssen, R. J. Stokroos, H. Kingma, R. van de Berg

**Affiliations:** ^1^Department of Otorhinolaryngology and Head and Neck Surgery, Maastricht University Medical Center, Maastricht, Netherlands; ^2^Department of Radiology, Maastricht University Medical Center, Maastricht, Netherlands; ^3^Department of Methodology and Statistics, School for Public Health and Primary Care (CAPHRI), Maastricht University, Maastricht, Netherlands; ^4^Faculty of Physics, Tomsk State University, Tomsk, Russian Federation

**Keywords:** Ménière’s disease, MRI, imaging, radiomics, quantitative image analysis, labyrinth, 3D models, vertigo

## Abstract

**Objective:**

The purpose of this exploratory study was to investigate whether a quantitative image analysis of the labyrinth in conventional magnetic resonance imaging (MRI) scans using a radiomics approach showed differences between patients with Ménière’s disease (MD) and the control group.

**Materials and methods:**

In this retrospective study, MRI scans of the affected labyrinths of 24 patients with MD were compared to the MRI scans of labyrinths of 29 patients with an idiopathic asymmetrical sensorineural hearing loss. The 1.5- and 3-T MRI scans had been previously made in a clinical setting between 2008 and 2015. 3D Slicer 4.4 was used to extract several substructures of the labyrinth. A quantitative analysis of the normalized radiomic image features was performed in Mathematica 10. The image features of the two groups were statistically compared.

**Results:**

For numerous image features, there was a statistically significant difference (*p*-value <0.05) between the MD group and the control group. The statistically significant differences in image features were localized in all the substructures of the labyrinth: 43 in the anterior semicircular canal, 10 in the vestibule, 22 in the cochlea, 12 in the posterior semicircular canal, 24 in the horizontal semicircular canal, 11 in the common crus, and 44 in the volume containing the reuniting duct. Furthermore, some figures contain vertical or horizontal bands (three or more statistically significant image features in the same image feature). Several bands were seen: 9 bands in the anterior semicircular canal, 1 band in the vestibule, 3 bands in the cochlea, 0 bands in the posterior semicircular canal, 5 bands in the horizontal semicircular canal, 3 bands in the common crus, and 10 bands in the volume containing the reuniting duct.

**Conclusion:**

In this exploratory study, several differences were found in image features between the MD group and the control group by using a quantitative radiomics approach on high resolution T2-weighted MRI scans of the labyrinth. Further research should be aimed at validating these results and translating them in a potential clinical diagnostic method to detect MD in MRI scans.

## Introduction

Ménière’s disease (MD) is a disorder of the inner ear, which is characterized by recurrent attacks of vertigo. These attacks are accompanied by a fluctuating sensorineural hearing loss and tinnitus or a sense of fullness in the affected ear (1). The exact cause and pathophysiology of MD is unclear and includes genetic, anatomic, metabolic, endocrine, autoimmune, vascular, allergic, viral, and traumatic factors (2). Histopathologic examination in patients with MD shows a distention of Reissner’s membrane (endolymphatic hydrops) in the cochlea or endolymphatic compartment of the labyrinth (3, 4). Since histopathologic examination (the gold standard) is not possible in a clinical setting, the diagnosis depends on the symptoms of MD (1, 5). For the diagnosis of definite MD, one of the criteria is that other causes of the symptoms have been excluded. Magnetic resonance imaging (MRI) is often indicated because of asymmetrical sensorineural hearing loss and to exclude other possible causes (6). However, it remains difficult to differentiate between MD and other causes of vertigo. Therefore, new imaging techniques are under investigation as a MD diagnostic, which include cone beam computed tomography (7) and MRI enhanced by invasive contrast agents such as gadolinium (8, 9). The administration of an intratympanic gadolinium injection is an invasive procedure and although adverse events are rare after intravenous contrast media, they are known to occur (10). A non-invasive imaging technique would be preferable as it could be argued that the invasiveness of this procedure does not justify the (potential) gain in diagnostic information it provides.

Here, an alternative approach is taken by analyzing quantitative data in conventional MRI scans. The evidence is increasing that with new imaging processing and analysis techniques, the evidence is increasing that with new imaging processing and analysis techniques, more information can be gathered from standard imaging modalities (11, 12). Texture analysis uses features such as the distribution of gray levels in an area or volume in an MRI scan (13). Radiomics refers to the extraction and analysis of such quantitative image features from medical images obtained with computed tomography (CT), positron emission tomography (PET), or MRI (14). These quantitative image features provide additional information about the analyzed structures that are not necessarily perceptually visible by the (neuro)radiologist. Another advantage is that standard-of-care images from the clinic can be used (14). In the process of radiomics, there are several steps to be followed (15). The first step is the acquisition of imaging (preferably standard-of-care). Second, an anatomical region has to be segmented to define the region of interest on the acquired image volume. In the study, here, this is the labyrinth. Third, quantitative image features have to be calculated from this segmentation (such as the mean of the intensities in the segmentation). Finally, these quantitative image features can be used for statistical analysis. Several recent studies have shown that radiomics can be used to obtain information about, for example, tumor phenotypes and prognosis (11, 12), tumor biomarkers (16), and distant metastasis (17). Studies have also shown structural differences in patients with amyotrophic lateral sclerosis (13) and Alzheimer’s disease (18) when compared to healthy subjects.

The purpose of this exploratory study was to investigate whether a quantitative image analysis of the labyrinth in conventional MRI scans using a radiomics approach showed differences between patients with MD and the control group.

## Materials and Methods

### Ethical Considerations

This study was performed in accordance with the guidelines outlined by Dutch legislation. According to the Medical Research Involving Human Subjects Act (WMO), ethical approval was not required due to the retrospective nature and anonymization of the data.

### Study Population

A retrospective study was performed. Patients with MD were identified from the patient registration system from a tertiary center. Patients with definite MD according to the criteria as accepted by the American Academy of Otolaryngology-Head and Neck Surgery (AAO-HNS) (1) were included, since these were the most recent criteria for MD during the inclusion period. In retrospect, the diagnostic criteria for definite MD as formulated in 2015 by the Bárány Society (5) also apply to all the patients with MD included in this study. Furthermore, an MRI scan of the cerebellopontine angle made in two preselected MRI scanners was required for inclusion. Since this retrospective study was performed in a tertiary center, many patients with MD had an MRI scan made in another center and could therefore not be included in this study. The labyrinth affected by MD was used in the study. In case of bilateral MD, one of the labyrinths was chosen. The exclusion criterion was motion artifacts on the MRI scan (the criterion was that the inner ear should be sharply delineated). The control group consisted of patients with idiopathic asymmetrical sensorineural hearing loss. These patients were chosen as controls, since this was a retrospective study and no MRI scans from people without hearing loss were available. The labyrinth least affected by hearing loss was included in the study, because this labyrinth was considered to be most representative for a healthy person. In the clinical setting, conventional clinical tests at the discretion of the ENT specialists were performed to diagnose the patients with an idiopathic asymmetrical sensorineural hearing loss. The exclusion criteria were composed of a documented history of vertigo or balance disorders and having motion artifacts on the MRI scan. The control group was matched to the MD group by scanning date to minimize biases which might arise from differences in scanning protocols over the years.

### MR Imaging

A Philips Intera MRI scanner (1.5 T) and a Philips Achieva MRI scanner (3 T) had been used to obtain the 3D High resolution T2-weighted images (Philips Nederland B.V., Eindhoven, The Netherlands). The scans had been previously made in a clinical setting between 2008 and 2015. The scan parameters were not constant, as can be seen in Table 1. All the scans had been previously evaluated by a radiologist.

**Table 1:** **Scan parameters**.

	1.5-T MRI scanner	3-T MRI scanner	
Scanning date	01-08-2012 until 17-05-2015	28-05-2008 until 31-05-2011	06-02-2013 until 26-04-2015
Repetition time (ms)	1500	2000	1500
Echo time (ms)	Between 169 and 182	200	Between 193 and 195
Slice thickness (mm)	0.6	1.0	0.8
Spacing between the slices (mm)	0.3	0.5	0.4
Echo train length	40	59	40
Magnetic field strength (T)	1.5	3	3

*Scan parameters and scanning data of the included MRI scans*.

### Image Extraction of the Labyrinth

**Figure 1:**
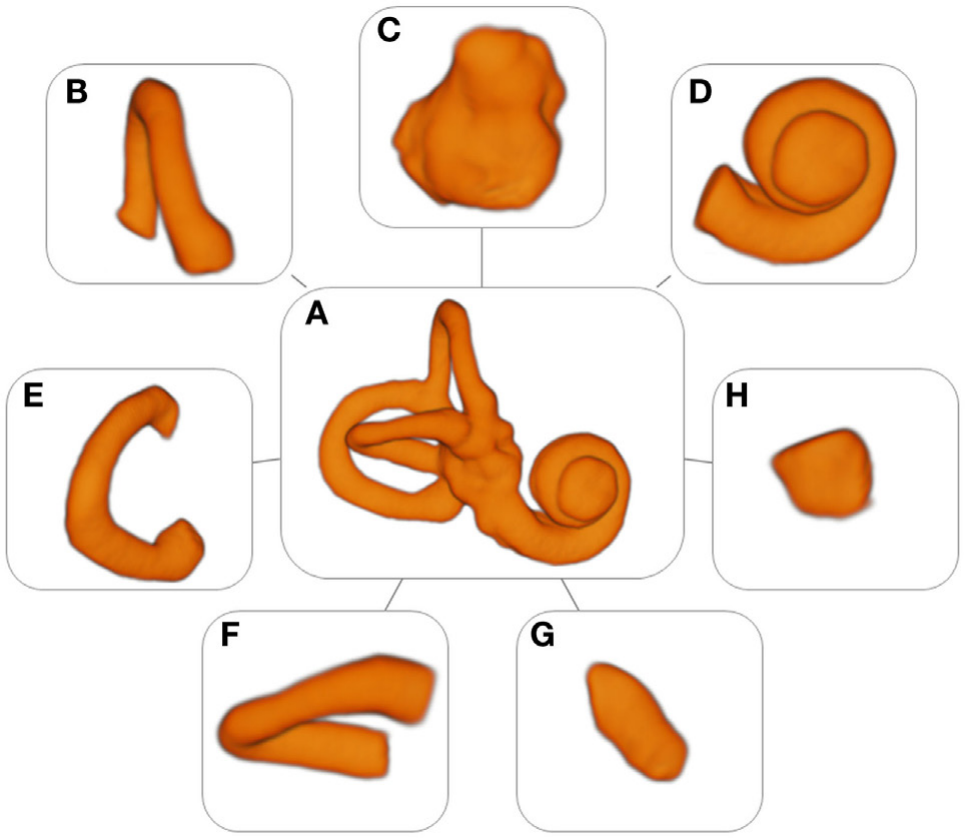
**Models of the labyrinth and its substructures**. **(A)** Labyrinth, **(B)** anterior semicircular canal, **(C)** vestibule, **(D)** cochlea, **(E)** posterior semicircular canal, **(F)** horizontal semicircular canal, **(G)** common crus, and **(H)** volume containing the reuniting duct.

3D Slicer 4.4, an open source software package for visualization and image analysis (19, 20) was used to extract the (sub)volumes from the MRI scans. First, a label was created in the shape of the labyrinth for the segmentation. The labyrinth was segmented into several substructures: the cochlea, the volume containing the reuniting duct, the vestibule, the semicircular canals, and the common crus (Figure 1). The labels were used as masks for intensity data extraction and to generate 3D models to measure the surface area and the volume of the different substructures and the labyrinth.

### Radiomic Feature Extraction and Statistical Analysis

Mathematica 10 (Wolfram Research, Champaign, IL, USA) was used for the radiomic feature extraction and statistical analysis. 3D models from the separate substructures were created with the edited MRI scans. These 3D models were modified by using 25 different image processing filters, resulting in a total of 26 primary radiomic features. A few examples are the entropy filter, the Laplacian filter, and the Fourier DCT filter. Every model was used to calculate 23 secondary radiomic features, which were statistical values such as the minimal intensity, the maximal intensity, and the mean intensity. The radiomic features were based on standard functions available in Mathematica 10. A list of different functions used can be found in Tables 2 and 3. In total, 598 image features were extracted. Because of differences between MRI scanners and scanning protocols, the image features were normalized. This was done by dividing the features from the different substructures of one patient by the features of the entire labyrinth of the same patient, thereby diminishing potential systematic differences introduced by different scanner’s and scanner protocols (Table 1). For the statistical analysis of the radiomic image features, a permutation test was used. A two-sided *p*-value of <0.05 was considered significant.

**Table 2:** **Primary radiomic features**.

1	No filter
2	FourierDCTFilter
3	EdgeDetect
4	GradientOrientationFilter
5	EntropyFilter (range 1)
6	EntropyFilter (range 2)
7	EntropyFilter (range 3)
8	EntropyFilter (range 4)
9	EntropyFilter (range 5)
10	EntropyFilter (range 6)
11	LaplacianFilter
12	RidgeFilter
13	LaplacianGaussianFilter
14	ClusteringComponents
15	MorphologicalComponents
16	MorphologicalBinarize
17	DiscreteWaveletTransform (1)
18	DiscreteWaveletTransform (2)
19	DiscreteWaveletTransform (3)
20	DiscreteWaveletTransform (4)
21	DiscreteWaveletTransform (5)
22	DiscreteWaveletTransform (6)
23	DiscreteWaveletTransform (7)
24	DiscreteWaveletTransform (8)
25	ImageSaliencyFilter
26	ColorToneMapping

*Functions used in Mathematica 10 (Wolfram Research, Champaign, IL, USA) to create the primary radiomic features*.

**Table 3:** **Secondary radiomic features**.

1	ImageMeasurements, MinIntensity
2	ImageMeasurements, MaxIntensity
3	ImageMeasurements, MeanIntensity
4	ImageMeasurements, MedianIntensity
5	ImageMeasurements, StandardDeviationIntensity
6	ImageMeasurements, TotalIntensity
7	ImageMeasurements, Skew
8	ImageMeasurements, IntensityCentroid (*x*-coordinate)
9	ImageMeasurements, IntensityCentroid (*y*-coordinate)
10	ImageMeasurements, IntensityCentroid (*z*-coordinate)
11	ImageMeasurements, Entropy
12	ImageMeasurements, Energy
13	DominantColors (amount)
14	DominantColors (primary)
15	VarianceCI (low)
16	VarianceCI (high)
17	Kurtosis
18	TrimmedMean
19	MeanDeviation
20	RootMeanSquare
21	Variance
22	Commonest (mean)
23	AutocorrelationTest

*Functions used in Mathematica 10 (Wolfram Research, Champaign, IL, USA) to create the secondary radiomic features*.

## Results

### Ménière’s Disease Group and Control Group

In total, 24 patients who met the inclusion criteria for MD were included in this study. The control group consisted of 29 patients. The two groups were similar with regard to age, the labyrinth analyzed, and distribution between MRI scanners and scanning dates (Table 4). More men than women were included in both groups. The median hearing loss in the analyzed ear was higher in the MD group than in the control group.

**Table 4:** **Patient demographics**.

		Ménière’s disease group (*n* = 24)	Control group (*n* = 29)
Age at the moment of scanning in years [median (interquartile range)]		61 (51–71)	56 (48.8–64.3)
Gender (F/M)		37.5/62.5%	13.8/86.2%
Analyzed labyrinth (left/right)		50.0/50.0%	37.9/62.1%
MRI-scanner (1.5/3 T)		41.7/58.3%	34.5/65.5%
Scanning date (range)		29-10-2008 until 17-05-2015	28-05-2008 until 26-04-2015
Distribution of scanning dates	2008	4.2%	3.5%
	2009	4.2%	3.5%
	2010	4.2%	3.5%
	2011	4.2%	3.5%
	2012	12.5%	20.7%
	2013	25.0%	48.3%
	2014	33.3%	10.3%
	2015	12.5%	6.9%
Average hearing loss^a^ in analyzed ear in dB [median (interquartile range)]		61 (42–75)	13 (8–25.5)
Bilateral MD (yes/no)		20.8/79.2%	–

*Information about the Ménière’s disease group and the control group*.

*^a^The audiogram closest to the date of scanning was used*.

### Surface Area and Volume

The surface area and volume of the labyrinth and its separate substructures were not significantly different between the two groups (Table 5).

**Table 5:** **Surface area and volume of the labyrinth and its separate substructures**.

		Ménière’s disease (*n* = 24)	Control group (*n* = 29)	*p*-Value
Surface area in mm^2^ [median (interquartile range)]	Cochlea	135.29 (129.08–146.86)	144.31 (135.91–150.45)	0.122
	Volume containing the reuniting duct	31.06 (25.64–35.14)	27.72 (25.19–31.78)	0.195
	Vestibule	95.39 (91.97–104.12)	95.43 (89.78–107.69)	0.721
	Anterior semicircular canal	70.03 (63.34–74.12)	68.82 (61.25–77.39)	0.514
	Posterior semicircular canal	79.48 (70.77–86.47)	82.74 (71.33–90.04)	0.574
	Horizontal semicircular canal	61.23 (50.92–68.34)	58.89 (50.48–65.90)	0.688
	Common crus	21.38 (18.86–23.78)	23.44 (19.85–25.82)	0.284
	Labyrinth	435.63 (412.51–478.74)	442.97 (413.05–473.11)	0.649
Volume in mm^3^ [median (interquartile range)]	Cochlea	80.22 (74.96–90.51)	86.80 (76.04–97.29)	0.335
	Volume containing the reuniting duct	12.82 (9.47–14.86)	10.53 (9.16–12.86)	0.172
	Vestibule	65.27 (60.04–72.31)	65.21 (58.07–76.90)	0.957
	Anterior semicircular canal	22.73 (17.65–26.32)	20.71 (17.00–25.10)	0.437
	Posterior semicircular canal	26.49 (21.43–29.49)	27.63 (20.16–31.04)	0.979
	Horizontal semicircular canal	20.42 (14.03–23.78)	18.19 (13.38–21.66)	0.469
	Common crus	7.01 (5.61–8.21)	8.14 (5.84–9.15)	0.348
	Labyrinth	236.26 (202.02–272.98)	239.68 (207.96–267.81)	0.936

*The surface area and volume of the labyrinth and its separate substructures*.

### Models

A set of 53 detailed models of the labyrinth and its separate substructures were formed (Figure 1). These models were used to extract the radiomic image features. In addition, they provided an accurate three-dimensional image of the labyrinth and its substructures.

### Radiomic Image Features

**Figure 2:**
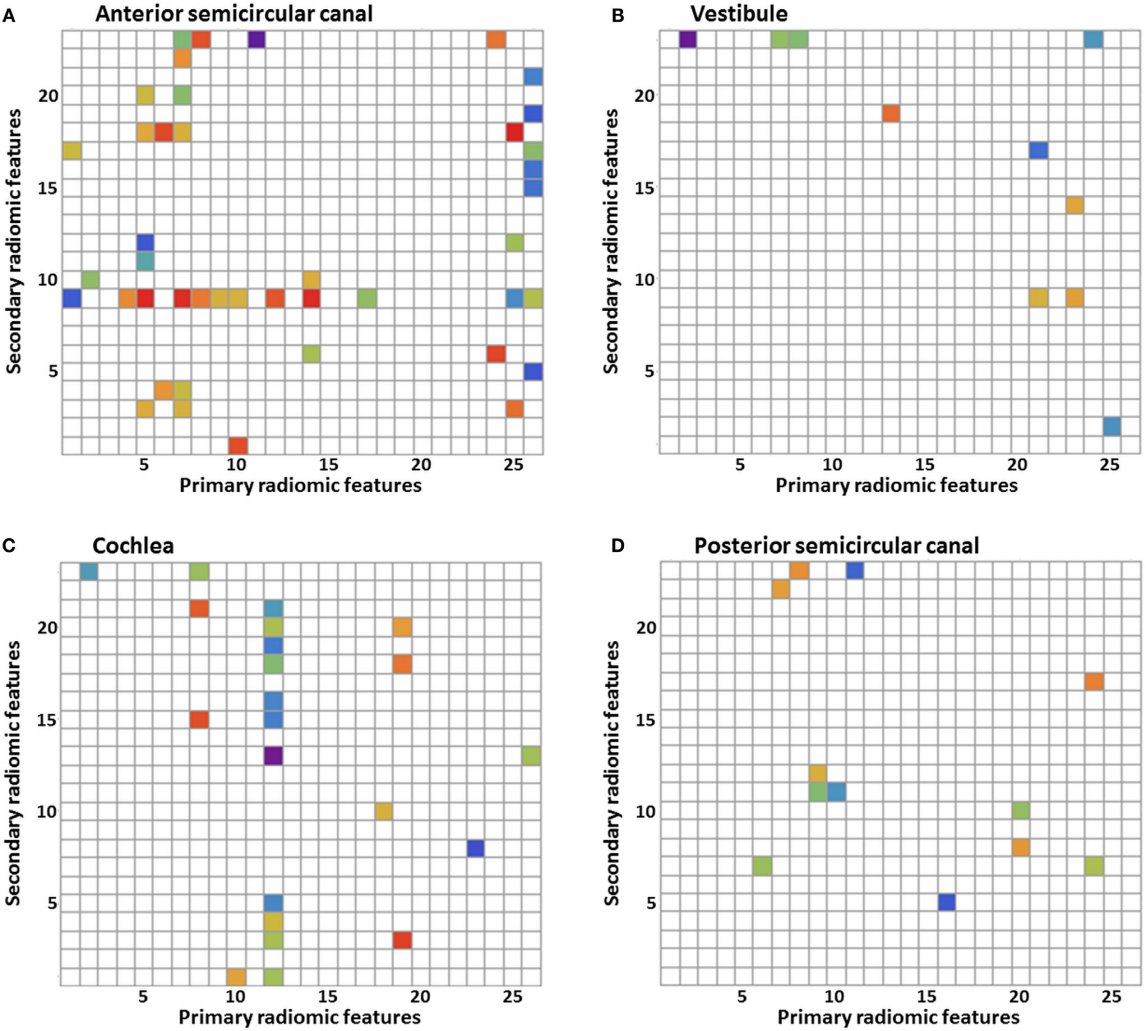

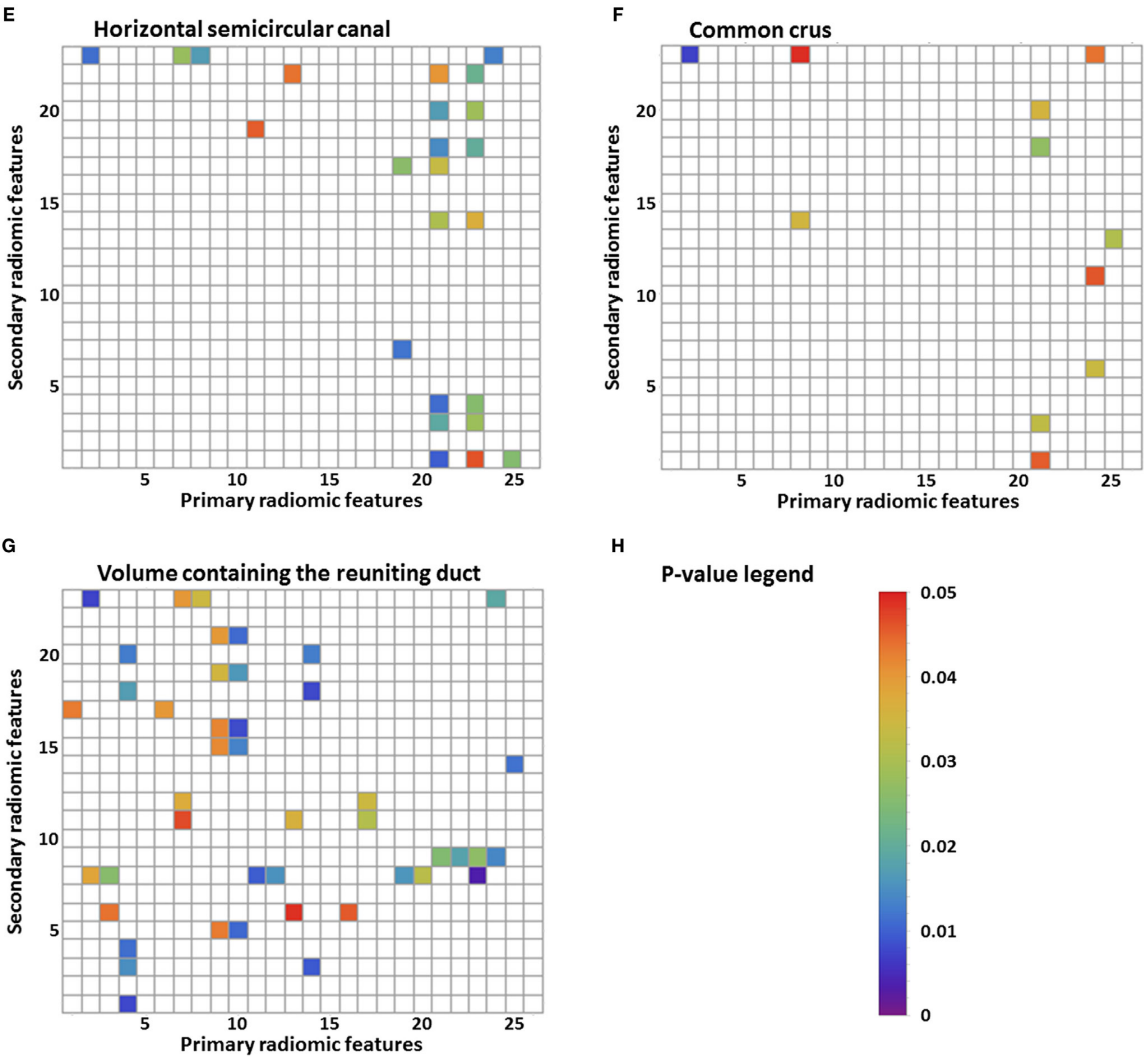
**Results of the statistical analysis of the radiomic image features**. Every *p*-value <0.05 is shown in color. Primary radiomic features are shown on the *x*-axis; these represent the different image processing filters. The secondary radiomic features are shown on the *y*-axis; these are the statistical values calculated from the primary radiomic features. A list of the primary radiomic features can be found in Table 2 and a list of the secondary radiomic features can be found in Table 3. A vertical band means that there are several significant image features in one primary radiomic feature, and a horizontal band means that there are several significant image features in one secondary radiomic feature. The figures represent one of the substructures of the labyrinth: **(A)** anterior semicircular canal, **(B)** vestibule, **(C)** cochlea, **(D)** posterior semicircular canal, **(E)** horizontal semicircular canal, **(F)** common crus, **(G)** volume containing the reuniting duct, and **(H)** *p*-value legend.

A total of 598 radiomic image features were analyzed in every substructure of the labyrinth. In all the separate substructures, statistically significant differences between the MD group and the control group were seen in several image features (Figures 2A–G). Figure 2H shows the p-value legend. In the figures, differences are seen between the amount of statistically significant image features in the different substructures: 43 in the anterior semicircular canal, 10 in the vestibule, 22 in the cochlea, 12 in the posterior semicircular canal, 24 in the horizontal semicircular canal, 11 in the common crus, and 44 in the volume containing the reuniting duct. For example, there are more imaging features that resulted in statistically significance in the volume containing the reuniting duct and in the anterior semicircular canal than in the other substructures. Furthermore, some figures contain vertical or horizontal bands, which show that there are several image features statistically significant in the same primary or secondary image feature. We have defined a band as three or more statistically significant image features in the same row or column. For example, Figure 2C shows a vertical band in the twelfth primary radiomic feature, and Figure 2A shows a horizontal band in the ninth secondary radiomic feature. In Figures 2A–G, several bands are seen: 9 bands in the anterior semicircular canal, 1 band in the vestibule, 3 bands in the cochlea, 0 bands in the posterior semicircular canal, 5 bands in the horizontal semicircular canal, 3 bands in the common crus, and 10 bands in the volume containing the reuniting duct.

## Discussion

Significant differences in radiomics image features between the MD group and the control group were found in all the substructures of the labyrinth. The band-like patterns shown in Figures 2A–G are more important than just the significant *p*-values as its interpretation is less at risk for chance findings because no corrections for multiple testing were performed. These bands indicate that primary or secondary radiomic modifications amplify features with a certain consistency. The differences found in the substructures do not necessarily reflect visually perceptible differences. Research has been performed into the histopathology of MD (4, 21) also using proteomic techniques (22) and qualitative imaging techniques (7). This might provide an explanation for the observed differences in image features. The histopathology observed in a study of the temporal bones of patients with MD is endolymphatic hydrops (4). A distension, herniation, and rupture of the endolymph compartment were not necessarily confined to the cochlea. Pathologic changes were found to be present in the whole labyrinth, though the canals and common crus were affected the least (21). In another study, it was suggested that an inflammatory or autoimmune reaction in the inner ear may cause damage to the epithelial layers surrounding the endolymphatic space (22). Results found in this study correspond with these findings, since a significant difference was found in all the substructures of the labyrinth. However, a meta-analysis of temporal bone reports showed that the lesion distribution was orderly from the cochlea to the saccule, utricle, ampullae, and then the canal system (21). In our study, we did not find this order, since there were more statistically significant image features in, for example, the anterior semicircular canal than in the cochlea. The difference in image features could hypothetically be explained by the damage to or different distribution of differently charged fluids in several parts of the labyrinth, causing a different distribution of the intensities. A recent study using a proteomic approach found differences in protein composition of the inner ear fluid in MD patients in comparison to controls. The inner ear fluid of MD patients contained other immunoglobulins and its variants (22). Since γ-globulin has a higher relaxivity in serum (23), it could be a possible explanation for the differences in image features found in this study. Another study found a significant difference in the shape of the reuniting duct using cone beam CT (7). In this study, more imaging features that resulted in statistically significance were found in the volume containing the reuniting duct than in the other substructures, but this volume was also considerably smaller increasing the risk on a chance finding.

### Future Clinical Implementation

The next step in exploring the application of radiomics for diagnosing patients with MD is to determine the accuracy as a diagnostic tool. For this purpose, an extensive internal and external validation is necessary. Recent research demonstrated that machine learning and radiomics can be used to predict overall survival in lung cancer patients (24). To investigate whether machine learning could be used for diagnosing MD patients, it should be trained and validated on two separate sets of data. The use of an independent test set would provide information on the diagnostic accuracy of this method. The advantage of using machine learning in combination with radiomics is that the analysis of the labyrinth could possibly be done autonomously as automatic image segmentation and registration are already feasible (25). The scalability that results from such a setup could possibly reshape diagnostics of the inner ear in the field of (neuro)radiology. In addition, it might be possible to identify patients who for some reason are difficult to diagnose by using the criteria for definite MD, for example, because of fluctuating hearing loss.

**Figure 3:**
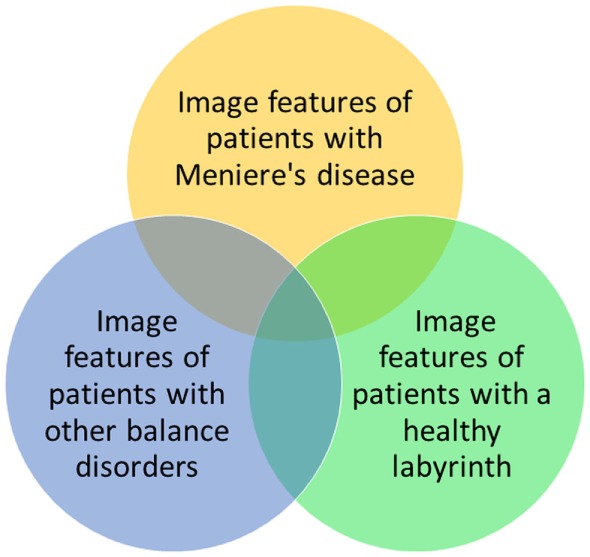
**Possible relationships between image features of patients with Ménière’s disease, patients with other balance disorders, and patients with a healthy labyrinth**. In this study, the yellow and green groups are investigated.

These results are promising, but radiomics cannot be used in the diagnosis of patients with MD yet. MD shows several gradations that are not yet distinguished objectively but possibly could be in the future by performing a cluster analysis on image features. Also in clinical practice, it is necessary to differentiate between three populations: patients with MD, patients with other balance disorders, and patients with complaints of dizziness which cannot be objectified (yet) (Figure 3). To enhance the differentiation between these groups, it could be helpful to cluster the relevant features to form a more robust model. Furthermore, during the last several years, several studies have been performed into the visualization of endolymphatic hydrops by using intravenous or intratympanic contrast agents such as gadolinium (8, 9, 26). Studies have been performed that have proposed a grading system for endolymphatic hydrops on MRI (27, 28) or that found a correlation between the progression of disease and endolymphatic hydrops on MRI (29). Further research is necessary to compare the radiomics method to the use of MRI enhanced by contrast agents, to see if similar results are found. By clustering several image features, the feasibility of a grading system in the radiomics method or a correlation between the progression of disease or progression of hearing loss can also be explored. The clustering of features can possibly also be used to separate hitherto unidentified subtypes of MD. Another advantage of the radiomics method is that no invasive contrast agents are necessary.

### Limitations

A limitation in this study is that it did not include other balance disorders or other otologic conditions that could alter the composition of the labyrinth. The aim of this exploratory study instead was to investigate whether radiomic image features allow for differences to be detected between patients with MD and controls on conventional MRI scans and to provide a proof of concept for this method. Furthermore, since the aim was not to identify a single feature that could be used to diagnose a patient with MD but to identify features that could in the future be combined and used for subsequent machine learning, no correction for multiple testing was performed. Doing so would minimize false positives but could also obscure potentially important features. The statistical significant results could therefore include false positives.

Despite having two similar groups in terms of demographics (Table 4) and surface area and volume of the substructures (Table 5), confounding differences concerning the MRI scanner setup cannot be completely ruled out in this relatively small sample size (Table 1). To minimize the effect of different scan parameters, the control group was matched to the MD group by scanning date. Furthermore, by normalizing the values, it was deemed possible to compare the data, because relative differences could be analyzed. On the other hand, the variability in MRI scanners in this study might also be an advantage. If radiomics could be used clinically for the diagnosis of MD in the future, it could be more widely implemented if the method is robust and independent of the type of MRI scanner or scanning parameters. Furthermore, we have included both unilateral and bilateral MD patients in this study, seeing that in the future the model would be more robust if both types of MD were included. Even though the etiopathogenesis might not be the same, the structural differences in the labyrinth are thought to be similar (21) and both show endolymhatic hydrops on MRI enhanced by contrast agents (30).

## Conclusion

In this exploratory study, several differences were found in image features between the MD group and the control group by using a quantitative radiomics approach on high resolution T2-weighted MRI scans of the labyrinth. Further research should be aimed at validating these results and translating them in a potential clinical diagnostic method to detect MD in MRI scans.

## Author Contributions

All the authors contributed to the design of the work presented in this paper. EB and MH designed the experiment, gathered the data, performed the analysis, and wrote the manuscript. RB designed the experiment, performed the analysis, supervised the writing, reviewed the manuscript, and edited the manuscript. AP and AJ performed the analysis, reviewed the manuscript, and edited the manuscript. RS and HK reviewed the manuscript and edited the manuscript. All the authors take full responsibility for the correctness of this paper and approved the final version.

## Conflict of Interest Statement

The authors declare that the research was conducted in the absence of any commercial or financial relationships that could be construed as a potential conflict of interest.
